# Oscillatory Dynamics Track Motor Performance Improvement in Human Cortex

**DOI:** 10.1371/journal.pone.0089576

**Published:** 2014-02-27

**Authors:** Stefan Dürschmid, Fanny Quandt, Ulrike M. Krämer, Hermann Hinrichs, Hans-Jochen Heinze, Reinhard Schulz, Heinz Pannek, Edward F. Chang, Robert T. Knight

**Affiliations:** 1 Department of Neurology, Otto-von-Guericke University, Magdeburg, Germany; 2 Leibniz Institute of Neurobiology (LIN), Magdeburg, Germany; 3 Department of Neurology, University of Lübeck, Lübeck, Germany; 4 German Center for Neurodegenerative Diseases (DZNE), Magdeburg, Germany; 5 Epilepsiezentrum Bethel, Bielefeld, Germany; 6 Department of Neurological Surgery, University of California San Francisco, San Francisco, California, United States of America; 7 Helen Wills Neuroscience Institute, University of California, Berkeley, California, United States of America; 8 Department of Psychology, University of California, Berkeley, California, United States of America; The University of Western Ontario, Canada

## Abstract

Improving performance in motor skill acquisition is proposed to be supported by tuning of neural networks. To address this issue we investigated changes of phase-amplitude cross-frequency coupling (paCFC) in neuronal networks during motor performance improvement. We recorded intracranially from subdural electrodes (electrocorticogram; ECoG) from 6 patients who learned 3 distinct motor tasks requiring coordination of finger movements with an external cue (serial response task, auditory motor coordination task, go/no-go). Performance improved in all subjects and all tasks during the first block and plateaued in subsequent blocks. Performance improvement was paralled by increasing neural changes in the trial-to-trial paCFC between theta (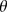; 4–8 Hz) phase and high gamma (HG; 80–180 Hz) amplitude. Electrodes showing this covariation pattern (Pearson's r ranging up to .45) were located contralateral to the limb performing the task and were observed predominantly in motor brain regions. We observed stable paCFC when task performance asymptoted. Our results indicate that motor performance improvement is accompanied by adjustments in the dynamics and topology of neuronal network interactions in the 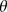 and HG range. The location of the involved electrodes suggests that oscillatory dynamics in motor cortices support performance improvement with practice.

## Introduction

Phase-amplitude cross-frequency coupling (paCFC) of oscillations in different frequency bands has been proposed as an effective mechanism to form functional networks that recruit local neuronal populations across a global spatial scale [1]–[4]. Phase-amplitude CFC between HG (80–150 Hz) amplitude to 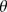 (4–8 Hz) phase was first described by [2] and later confirmed by other authors in rats [5], [6] and humans [7]. During paCFC amplitudes of higher frequency oscillations, reflecting local cortical processing, are modulated by the phase of low frequency oscillations [8]–[12]. This mechanism has been proposed to engage and coordinate local processing modules across spatially distributed brain areas supporting cognition and motor performance [4], [9], [13]–[17].Further support for this proposal comes from recent clinical studies linking altered paCFC to debilitating psychiatric and motor disorders [18]–[21]. Moreover, paCFC is prominent during language and motor tasks [2], [4] and the frequency of the slower phase coupling oscillation is task dependent [12]. However, beyond clinical studies evidence for a functional role of paCFC in the process of organizing human cognition and behavior is limited predominantly to the memory domain (see [22] for a review). Axmacher and colleagues [23] reported that inter-individual differences in working memory performance correlated with differences in paCFC precision, supporting the functional relevance of CFC for memory processing. Tort and colleagues [6] examined the dynamic modification of functional relations between performance and CFC in rat hippocampus and found coupling strength between 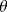 and gamma (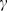: 25–100 Hz) correlated with maze learning.

A stronger link between paCFC and behavior in humans would be supported by a correlation between paCFC and trial-by-trial variations in performance. To address this, we examined the relation between paCFC and motor performance improvement. We recorded the electrocorticogram (ECoG) in human patients (N = 6; mean age  = 20.5, std  = 5.5; 2 female) undergoing epilepsy diagnosis while they learned skilled motor behaviors. To assess the link between paCFC and behavior we compared changes in paCFC to changes in performance over an extended time scale during motor skill acquisition, and correlated performance and paCFC at the single trial level. We show that paCFC in intracranial subdural recordings between 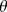 (4–8 Hz) and HG (80–180 Hz) in the human cortex tracks level of motor performance across different motor tasks.

## Results

### Phase amplitude cross frequency coupling

**Figure 1:**
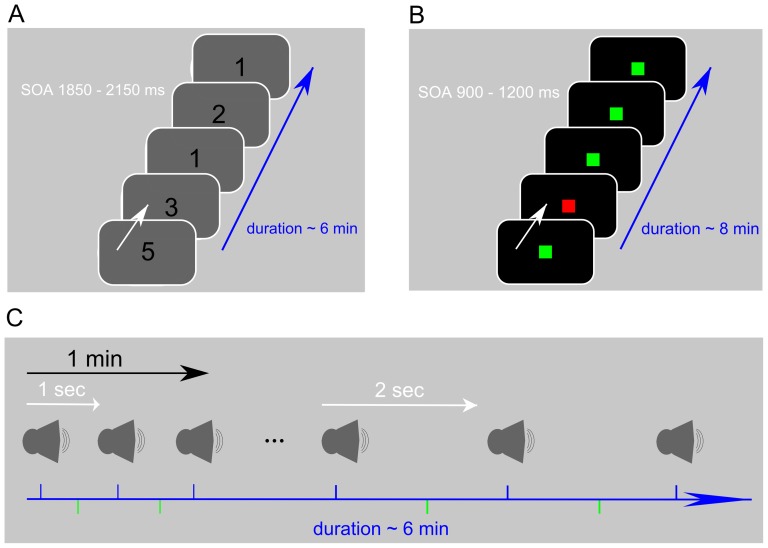
Paradigms employed (details described in Methods). A) Serial reaction time task: The numbers on the screen indicate the finger to be used for the key press. B) Go/no-go: Green indicates a go and red indicates a no-go trial. C) Auditory motor coordination: Subjects were instructed to press a key in the middle of the interval between two consecutive tones. The interval length was either one second or two seconds and was held fixed for one minute. Each subject carried out two blocks (see Methods).

**Figure 2:**
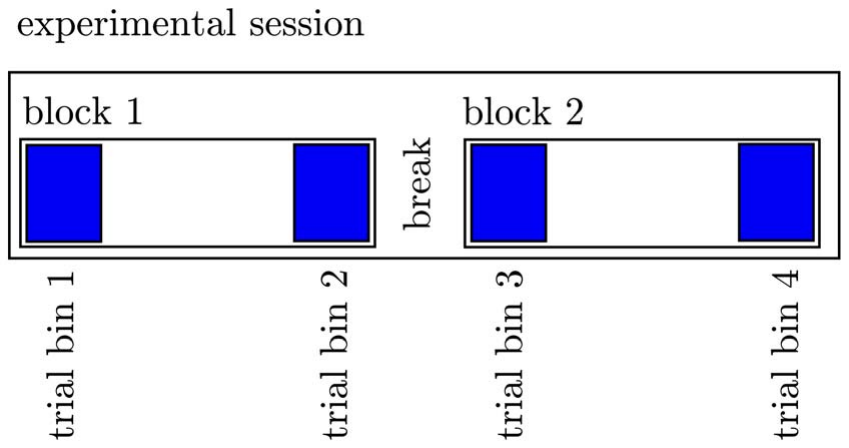
Here we depict the separation of the whole experimental session into trial bins. The experimental session in each patient consisted of 2 blocks separated by a short break. In each block we defined two trial bins each containing 30 trials (blue). We compared the PLV across the four trial bins to assess the evolution of connectivity length of and HG activity during motor performance improvement.

We investigated potential links between paCFC and motor performance in six subjects each performing one of three repetitive motor tasks described next. The three different behavioral tasks (Figure 1) were a serial reaction time task (SRT 2 subjects), a go/no-go task (GNG 3 subjects), and an auditory motor coordination task (AMCT 1 subject). All three tasks required the coordination of finger movements with an external stimulus. We assessed motor performance as reaction time in the SRT and GNG tasks and as the temporal deviation from the target time point in the AMC task. The cognitive requirements for performance improvement are different in all three tasks: learning the motor sequence in the SRT, learning the stimulus-response association in the GNG and improving movement timing in the AMCT. However, the motor component is performance improvement with practice. The dynamics of the different performance measures were assessed in a group statistic, by comparing the average behavioral outcomes between fixed trial bins (see Figure 2). We recorded the ECoG while subjects performed two blocks of one of each task with the hand contralateral to the electrode grid. The ECoG-time series were filtered in the 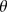 -band (4–8 Hz) and in the HG-band (80–180 Hz) yielding two separate filtered signals (see Methods). We calculated the analytic amplitude of the HG-band time series by taking the absolute value of the Hilbert transform of the filtered time series. The analytic amplitude is a new time series representing the amplitude envelope of the HG-oscillations at any moment in time. We performed the analysis on the 500 ms interval immediately following the stimulus onset. This interval includes the preparation of the responses indicated by the stimulus and includes approximately three 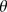-cycles.

**Figure 3:**
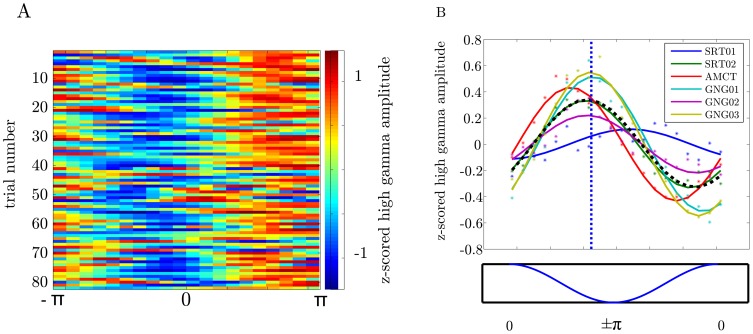
The amplitude of the HG oscillations is phase coupled to the 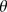 -band (4–8 Hz) oscillations in all subjects across paradigms. A) Time courses of sine wave functions fitted to the single trial amplitude envelopes of the HG oscillations of one subjects collapsed over electrodes. B) Sine wave functions fitted to the trial-averaged HG oscillation amplitudes envelopes of each subject. Each solid line represents the fit for one subject. Each dot represents the individual trial average of the HG oscillation in one of 20 intervals equally spaced over a 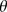 cycle. The black dashed line shows the averaged sine waves across subjects. The vertical blue dashed line denotes the averaged phase angle the HG amplitude peaks across subjects. The maximum of the 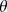 cycle is at phase 0 and the minimum at 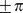.

We first asked whether the amplitude envelope of the local HG oscillations is phase coupled to the local 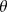-band oscillations. Figure 3A shows the time course of sine waves fitted to the single trial variations of HG analytic amplitude pooled across all electrodes in one subject. As predicted, HG analytic amplitude varied systematically over the 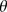-cycle. Figure 3B shows single subject sine wave fits to the HG analytic amplitude averaged over trials and electrodes. Each fit was significant (p
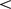
0.001) and the HG amplitude variations were consistent over subjects with only a slight deviation of subject 1 (see Figure 3B). The frequencies of the fitted sine waves are in the 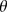 band (.95 Hz, SE: .02 Hz) and the phase angle is .6 rad (SE: .23 rad, see Methods for an explanation of the sine wave parameters - frequency and phase angle). The maximum of the HG-analytic amplitude centers around the trough in the 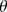-cycle (mean  =  2.56 rad, std  =  .56 rad, skewness  =  −.16).

### Covariation of paCFC with performance improvement

**Figure 4:**
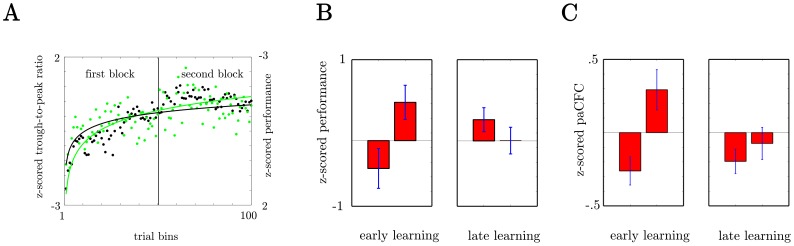
Covariation of average paCFC with performance over the time course of the experiment. A) The development of TPR and motor performance during the time course of the experiment. Results are collapsed across all six subjects/three experiments. Data for the first block and second block are shown in the left and second half of the plot. Each point represents the average in one of 100 time bins. Exponential functions fitted to the data z-scored over both blocks indicate a similar time course for performance and TPR. B) Subject averaged motor performance during the first and last sets of 30 trials in the first (early learning) and the second (late learning) experimental block. C) Subject averaged TPR. Data was z-scored within blocks and TPR was averaged over all electrodes.

To investigate if cross frequency coupling (paCFC) covaries with motor performance variations, we first calculated the trough to peak ratio (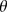-trough to HG peak ratio - TPR; see Methods) over all electrodes on the grids as a metric for paCFC and related it to behavioral performance. Figure 4A shows the development of TPR and motor performance over the time course of the two experimental blocks each subject completed. Both TPR and performance increased during the experiment, as indicated by the fitted exponential functions. A statistical test confirmed this finding. In this test, we first compared average motor performance in the initial 30 trials of the first block with performance in the final 30 trials and found a significant improvement (Wilcoxon rank sum test across all subjects: p 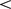 .05, Figure 4B, See Figure 2 for the structure of the experimental session and Table 1 for mean performance measures for each trial bin). However, performance plateaued in the second block as indicated by no significant difference (p = 0.18). The difference between the first and the second block is indicated by a significant block-by-trial-bin interaction in a two way ANOVA across subjects (F(1,20)  = 11.28; p = .003, df_error_  =  (Nsubj -1)*Ntrialbin). The next question was whether TPR exhibits the same behavior (Figure 4C). In concordance with behavioral performance we found, that the TPR increased between the first and the last 30 trials of the first block (Wilcoxon rank sum test across all subjects: p 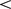 .05) but did not change between the first and the last 30 trials of the second block (p = 0.3). A significant block-by-trial-bin interaction in a two way ANOVA (F(1,20)  = 5.95; p = .03) confirmed that TPR changed during the first block and plateaued during the second block. This suggests that, on average, paCFC covaries with motor performance with paCFC and motor performance increasing early in the first experimental block and both plateauing in the second block.

**Table 1:** Behavioral data.

Paradigm		trial bin			
	Patient	1	2	3	4
SRT					
	SRT01	917 (271)	767 (227)	748 (219)	707 (161)
	SRT02	1472 (300)	1017 (284)	859 (207)	966 (232)
AMCT					
	AMCT01	117 (66)	98 (65)	147 (197)	142 (129)
Go/No-Go					
	GNG01	343 (46)	323 (117)	331 (109)	302 (128)
	GNG02	426 (185)	301 (42)	260 (28)	301 (66)
	GNG03	433 (168)	379 (178)	286 (40)	393 (89)

For SRT (serial reaction time task) and GNG (Go/No-Go) task reaction time is shown (standard deviation) in msec. For AMCT (auditory-motor coordination task) the absolute deviation from precision is shown also in msec. Each trialbin encompasses 30 trials.

**Figure 5:**
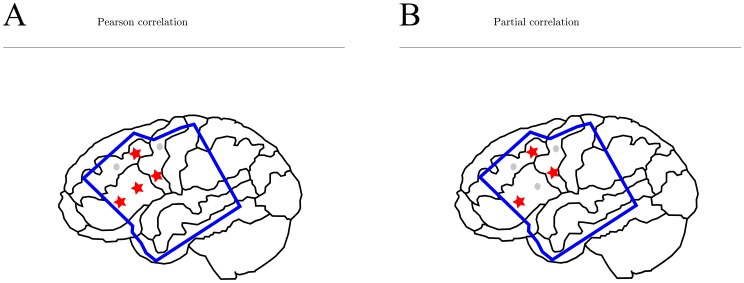
Depiction of the results from the ROI-analysis. A) ROIs with significant performance improvement unrelated TPR/performance correlations. B) ROIs with significant performance improvement related TPR/performance correlations. ROIs with significant correlations (Bonferroni correct for six comparisons) are marked with an asterisk. The 6 ROIs are the anterior and posterior medial frontal gyrus, the anterior and the posterior inferior frontal gyrus, and the superior and posterior sensorimotor cortex. The blue margin shows the grid coverage across all subjects with a square grid implanted.

Support for a functional relation between paCFC and motor performance would be provided by a trial-by-trial TPR with performance correlation. In order (i) to test for this correlation and (ii) to disentangle cortical regions showing varying paCFC with performance, we pooled the data in six anterior and frontal regions of interest (ROIs); the anterior and posterior medial frontal gyrus, the anterior and the posterior inferior frontal gyrus, and the superior and inferior sensorimotor cortex (see Figure 5) for all five subjects with a square 8×8 (N = 4)/16×16 (N = 1) grid implantation (see Figure S2). In each ROI we pooled the TPR values across electrodes and determined the p-values of the trial-by-trial correlation with performance of each ROI (Figure 5 for details see Methods and Figure S1). Significant correlation of TPR with motor performance (corrected for multiple comparisons) was observed in pre-/motor cortex and in anterior and posterior inferior frontal sulcus. We predicted two sources of variability in single trial correlation between TPR and motor paCFC: one that is performance improvement related and varies systematically over time and another one that is not related to performance improvement and varies randomly from trial to trial. The first analysis supported performance improvement related trial-by-trial correlations reflecting the co-evolution of coordination between brain networks and improvements of motor performance. We then calculated in the same ROIs the partial correlation of TPR with performance. This analysis factored out the fraction of correlation between TPR and performance which can be attributed to random trial-by-trial covariations and is performance improvement unrelated. This performance improvement unrelated correlation of TPR with motor performance was observed in sensorimotor cortex and in premotor cortex, in the posterior middle temporal sulcus (corrected for multiple comparisons) and overlaps with the performance improvement related correlation.

### Discrimination of performance improvement from Random Performance Fluctuations

**Figure 6:**
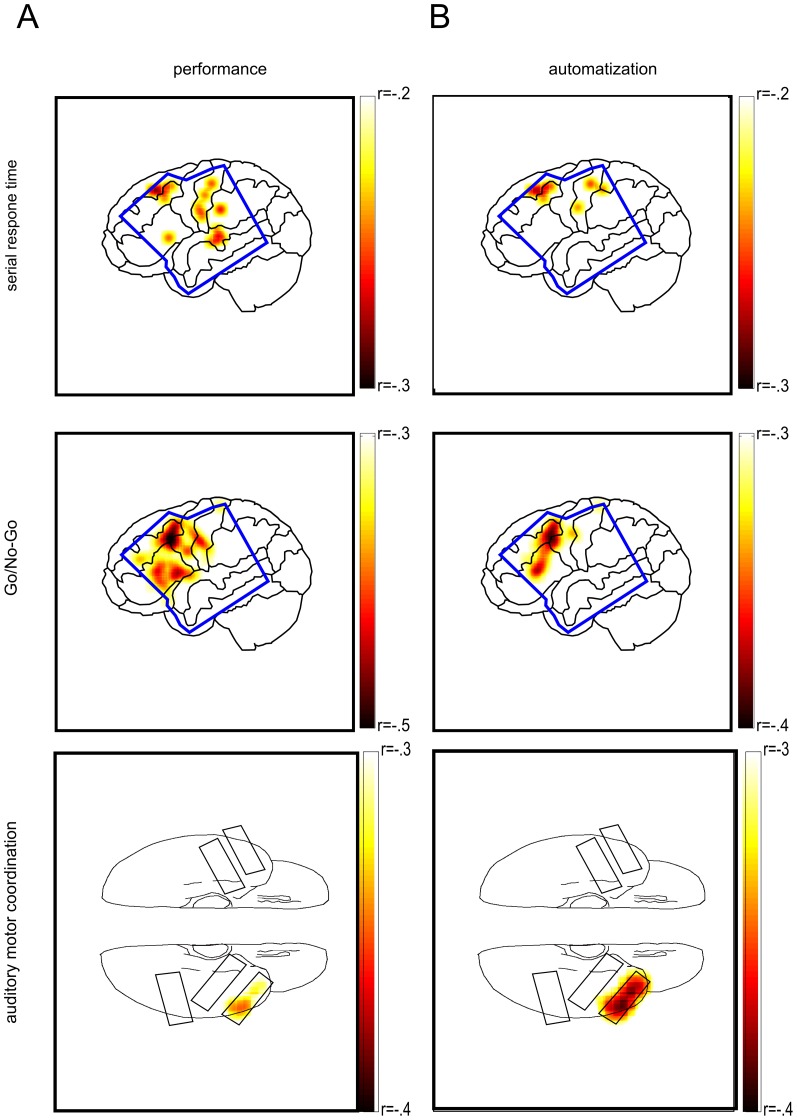
Electrodes with significant trial-by-trial correlations of TPR with performance. The significance threshold was determined in a permutation procedure (see Methods) A) Learning unrelated correlations of TPR with performance. B) Learning related correlations of TPR with performance. Darker colors indicate stronger correlations. See Methods for calculations on separating performance and learning related effects. The blue shape in the first and second row show the outline of all superimposed square grids. The black shapes in the third row denotes the grid locations for the participant in the AMCT. Spatial distortions result from the projection onto the cortex (for details see Figure S2).

To disentangle these two potential and functionally distinct causes of paCFC-performance covariations and disentangle the spatially wide ROIs we performed two different correlation analyses separately for each recording electrode. First, we calculated the partial correlation of TPR with performance. Second, we calculated the standard Pearson correlation between TPR and motor performance. Pearson correlation captured the performance improvement related plus the performance improvement unrelated correlation. Combined with partial correlation this was used to distinguish between the two effects. Electrodes that capture performance improvement related TPR with performance correlations should show a partial correlation different from zero and a Pearson correlation different from the partial correlation (see Methods). Importantly, we reasoned that if we observed a significant Pearson correlation in an electrode that significantly changes if we discount time related correlations (in partial correlation), then the TPR - performance correlation in this electrode is partly due to performance improvement related TPR - performance correlations. Figure 6A shows the electrodes where random trial-by-trial fluctuations of TPR correlated with motor performance (significant partial correlation - uncorrected for multiple comparisons). Clusters of electrodes showing high correlations are located in sensorimotor cortex, in premotor cortex, in lateral prefrontal cortex and in ventral anterior temporal cortex. Figure 6B shows the distribution of electrodes with performance improvement related trial-by-trial correlations between TPR and motor performance. Clusters of performance improvement related electrodes were apparent in premotor cortex, in lateral prefrontal cortex and in ventral anterior temporal cortex. Importantly, the variation of TPR with performance improvement was not a result of a shift of the HG amplitude peak relative to the 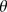 trough and hence the coupling phase remained stable during performance improvement (see Appendix S1).

## Discussion

Phase-amplitude cross-frequency coupling has been proposed to support interaction within functional networks [4]. Here we show that fluctuations of 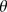 and HG paCFC are tightly linked to motor performance improvement at the single-trial level and show robust performance improvement clusters over pre-motor and motor cortices.

### Performance improvement and theta and high gamma activity

During motor performance improvement 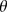 and HG activity show a clear development of coupling that asymptotes in strength as motor behavior performance improvement plateaus. paCFC is highly dynamic and task-specific [4], [24] and it has been proposed that paCFC enables adaptive behavior [2]. Here we report data from three different motor behavior experiments showing that paCFC dynamics reflect adaptive behavior supporting a relationship between paCFC and motor performance improvement on a trial-to-trial level. Notably, despite the differences in tasks similar cortical regions associated with performance improvement or random trial-to-trial performance are identified by paCFC evolution. The dynamic nature of paCFC and the task-specific coupling patterns have been shown in a variety of studies with task dependent differences in coupling frequencies and coupling phase [12], [24]–[27]. Here, we add an important paCFC characteristic. We show that even though the preferred phase as indicated by coupling phase stability does not change the activity pattern of both frequencies varies with behavioral changes.

### Biological mechanism

Oscillatory dynamics are proposed [1] to be inherent to the interplay of brain regions for cognitive control in memory and learning [21]. For example 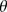 activity observed in hippocampal and neocortical regions varies as a function of the state of the subjects [28]. The neocortex exhibited more prominent 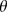 activity during wakefulness compared to REM sleep. Performance improvement during practice can be achieved by distributed 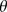 networks - which are up-regulated during wakefulness - by integrating or coordinating local activity. Here, the concept of information integration means that 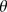 oscillatory activity accumulates and integrates the results of local processing as reflected in HG activity in the premotor/motor region. HG activity, either an indication of spiking activity or very fast network oscillations [25], [29], may be involved in planning and initiation of motor responses [30]. This frequency possibly reflects the activation of cortico-subcortical networks involved in the feedback control of discrete movements [31]. Taken together we speculate that information on planning of motor responses is integrated into memory by paCFC which results in performance improvement during the process of performance improvement.

### Conclusion

We identified cross-frequency coupling in the human cortex which is associated with motor performance variability per se. In this network a smaller area is integrated whose oscillatory dynamics reflect the progress in performance improvement. This learning related network suggests the establishment of a memory trace which is accumulated during practice and which is represented in a mutually adapted level of activity of 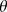 and HG activity [16], [32]–[34]. In this respect paCFC provides a mechanism subserving motor memory formation [2].

## Materials and Methods

### Patients

Six epilepsy patients undergoing pre-surgical monitoring with subdural electrodes participated in the experiments after providing their written informed consent. Experimental and clinical recordings were taken in parallel. Recordings took place at the University of California San Francisco (UCSF), CA, USA (4 Patients), Johns Hopkins University, Baltimore, USA (1 Patient) and the Epilepsy Center Bethel (ECB), Bielefeld (1 Patient), Germany and were approved by the local ethics committees (“Committee for the Protection of Human Subjects at UC Berkeley”, “Johns Hopkins Medicine Institutional Review Board” and “Ethical Committee of the University of Magdeburg”).

### Experimental Paradigms

We carried out three different motor tasks (serial reaction, go/no-go, auditory-motor coordination) with six different patients (Fig. 1). Each patient participated in one of the tasks. All paradigms required coordination of key presses on a computer keyboard to an external stimulus. Patients performed the task sitting upright in their bed using the hand contralateral to the grid.

#### Serial Reaction Task

The serial reaction task (SRT) consisted of a series of visually cued finger taps. The subjects had their fingers placed on different keys of a laptop keyboard (right hand: space bar, j, k, and ; - left hand: space bar, f, d, and a). Trials started with one of the numbers 1, 2, 3, or 5 appearing on a laptop-screen cueing the movement of thumb, index finger, middle finger, or little finger, respectively. Numbers were presented until a key was pressed but maximally for 2 seconds. In each subject the four numbers were presented in a fixed sequence (six items long) or random order depending of the block number but only fixed blocks were used. Each block took approximately 10 minutes. Two patients participated in this task (SRT01 - 02). They were instructed to press keys as fast and accurate as possible. One block took approximately six minutes.

#### Auditory-Motor Coordination Task

The second motor-paradigm was an auditory-motor coordination task (AMCT). One patient participated and was instructed to respond as accurately as possible halfway between successive auditory clicks presented at a constant rate. Seven click sequences, each 60 s long, were presented in a block. The inter-click-interval in a sequence was either 500 ms, 1000 ms or 2000 ms and the participant was informed that the interclick-interval changes. The seven click sequences with the differing interclick-interval were presented randomly within each block. Only sequences with a interclick-interval of 1000 and 2000 ms entered the analysis. The clicks were presented with speakers plugged into the laptop's sound card and placed in front of the patient at 1 m distance. One block lasted 7 min.

#### Go/No-Go task

Three patients participated in the go/no-go (GNG) task (GNG01 - 03). In each trial the patients were presented with either a green or red square of 100 ms duration and 900 - 1200 ms stimulus onset asynchrony. The subjects were instructed to respond as quickly as possible to green squares by pressing a key on a laptop keyboard but to withhold responses when a red square was presented (in block 1–3: 20% of the trials; in block 4: 50% of the trials). The participants were familiarized with the task in an initial short practice session. Only correct Go-trials of the first two blocks entered the analysis. Each block lasted approximately eight minutes.

### Data recording

At UCSF the electrocorticogram (ECoG) was recorded either from 64 platin-iridium-electrode grids arranged in an 8×8 array with 10 mm center-to-center spacing (FTT01, FTT02, GNG01, GNG02 GNG03) or from a 256 electrode grid (both Ad-Tech Medical Instrument Corporation, Racine, Wisconsin) arranged in a 16×16 array with 4 mm center-to-center spacing (GNG02). Exposed electrode diameter was 2.3 mm in the 64 electrodes grid and 1.8 mm in the 256 electrodes grid. The electrode signals were recorded with a 256 channel preamplifier (PZ2-256, Tucker-Davis Technologies (TDT), Inc) with the electrode furthest from the motor cortex used as a reference. The data from the pre-amplifier were sampled at 3051.7 Hz on a digital signal processor (RZ2 4 DSP, Tucker-Davis Technologies (TDT), Inc) with 16-bit resolution and stored to hard disk. Trigger signals indicating button presses and stimulus onsets were sent from the stimulus laptop via a USB-1208FS DAQ (Measurement Computing, Norton, MA) plus a photodiode attached to the screen and recorded on the DSP synchronized to the brain data. Trigger timing was additionally recorded on the stimulus laptop by querying the computers performance counter using the Psychophysics Toolbox (www.psychtoolbox.org). In Bielefeld (AMCT) the ECoG signal was recorded at 1000 Hz sampling frequency (16 Bit resolution) with a Nihon Kohden system (Tokyo, Japan) equipped with auxiliary analogue channels for synchronous recording of the trigger signals and the output from the sound card. Here 5 stripes were implanted each equipped with two parallel rows of 5 electrodes each (see Figure 6).

### Data analysis

**Figure 7:**
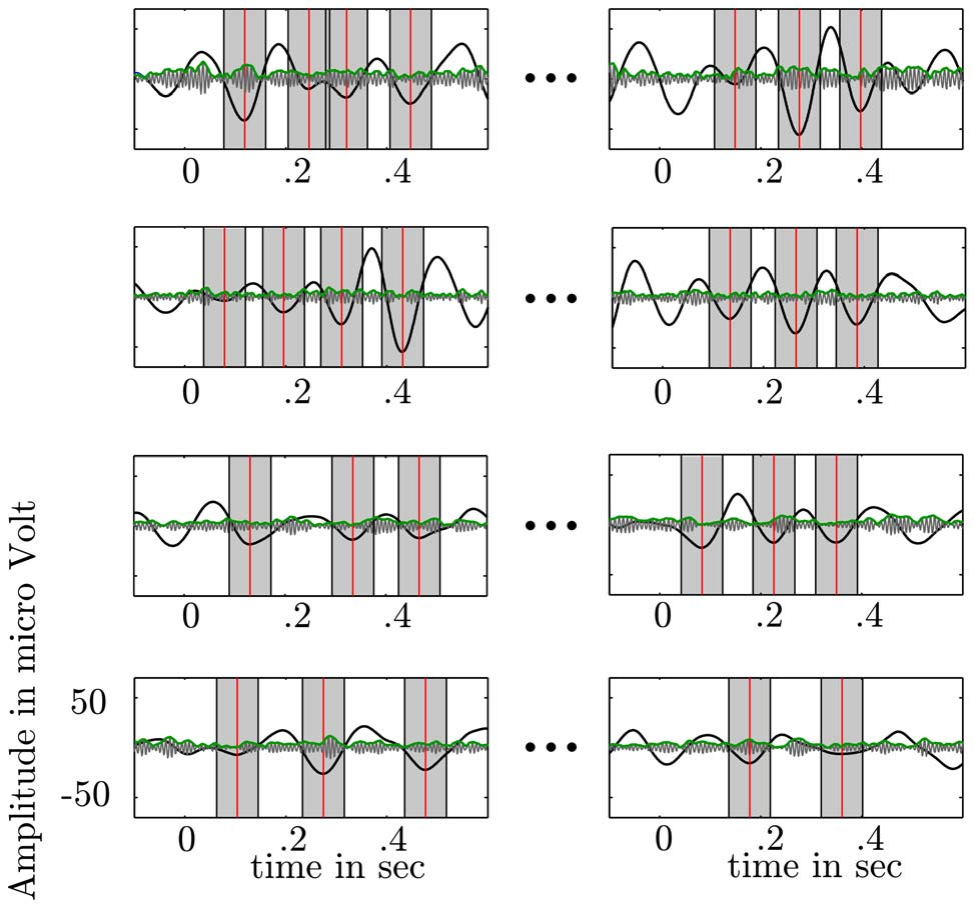
Calculation of the trough to peak ratio (TPR). We quantified paCFC as the ratio between 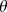 trough (local minima of the 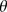 time series - red vertical lines) and HG amplitude at the corresponding 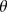 trough. Around each detected trough we spanned a window (half 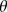 cycle - gray bars) in which 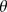 activity (black bold line) and HG amplitude (green line) was averaged.

We used Matlab 2008a (Mathworks, Natick, USA) for all offline data processing. We first preprocessed the recorded brain data and then we derived measures quantifying adaptation of oscillatory neural dynamics during motor skill learning. All filtering was done using IIR filters (Butterworth filter of order 4). Preprocessing served to remove non-physiological artifacts from the recorded data and to prepare them for further analysis. First we excluded channels exhibiting ictal activity or excessive noise from further analysis. In the remaining good channels we then excluded time intervals containing artifactual signal distortions such as steps of pulses by visual inspection. Finally, we re-referenced the remaining electrode time-series by subtracting the common average reference
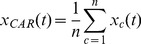
(1)calculated over the n good channels c from each channel time series. The resulting time series were then used to characterize brain dynamics over the time course of motor behavior performance improvement in terms of the TPR:

For each trial starting at stimulus onset we calculated the TPR to quantify the evolution of phase-amplitude cross-frequency interactions of cortical oscillations during motor skill learning. Therefore, we band-pass filtered each electrode's time series at two frequency bands, in the 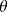-range (4–8 Hz) and in the HG (80–180 Hz) range since coupling was task relevant between these frequencies across a variety of experimental tasks [2]. We detected 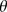-troughs, the local minima, in the 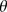-range filtered time series in the interval between 0 to 500 ms after stimulus onset (Figure 7). We obtained the HG analytic amplitude 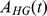 by Hilbert-transforming the HG filtered time series. For each detected 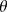-trough we then estimated the depth of the through 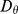 and the simultaneous HG amplitude as the average of the 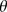-filtered and the 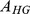 time series over an interval of 83 ms (half 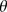 oscillation) centered on the trough. Note that multiple 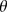 troughs fit into the 500 ms analysis leading to multiple estimates per trial. We averaged the individual estimates 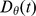 and 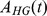 to obtain one measure for 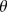 trough depth 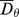 and one for HG amplitude 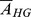 for each trial 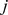. From these values we calculated TPR for each trial 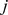 as:
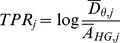
(2)


Taking the log of the ratio makes the distribution of TPRs symmetric. Note that the TPR includes both stimulus-locked and non-stimulus-locked brain activity. It summarizes the global cross frequency interaction on the grids.

### Phase-Amplitude Coupling

paCFC was tested by splitting the 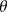 oscillations of the 500 ms analysis window into 20 equally spaced phase bins ranging from -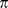 to 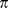 (18°or 0.314 rad) in each subject and each electrode. In each phase bin we averaged the amplitude envelope of the local HG. A cosine wave function
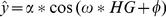
(3)with 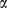 representing the amplitude, 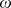 representing the frequency and 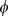 representing the phase angle was fitted to the resulting 20 HG amplitude (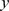) values. 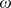 close to 1 indicates that HG amplitude variation is accounted for the 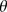 cycle.

### ROI analysis

In each patient we grouped electrodes according to the same anatomical landmarks in 6 regions of interest (see Figure S1): The anterior (in sum 34 electrodes across subjects) and posterior (44 electrodes) medial frontal gyrus, the anterior (49 electrodes) and the posterior inferior frontal gyrus (81 electrodes), and the superior (48 electrodes) and inferior (46 electrodes) sensorimotor cortex. We averaged the TPR-values within each ROI across electrodes. In each patient we determined the p-value for Pearson's correlation coefficient r and the partial correlation coefficient rho (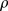) between the averaged TPR values and behavioral performance across trials. Each ROI in which the mean p-value across subjects fell below the p-value corrected for multiple comparisons (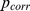  =  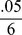) was considered statistically significant.

### Separating performance from learning effects

We separated performance from learning effects by applying a permutation test statistic. The reasoning for applying a permutation test was two-fold. First, we sought to correct the p-values for each electrode due to the many individual correlation tests applied. We tested this against a distribution which did not rely on the same temporal interval (500 ms following the stimulus presentation) for which the correlation coefficient was calculated. Second, we wanted to identify electrodes in which Pearsons correlation coefficient r was significantly higher than the partial correlation coefficient 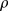. This means that we looked for electrodes with a significant difference between r and 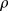. Since the significance can only be determined in relation to a distribution we estimated this distribution from our data. Hence, the null hypothesis to be rejected was that the difference between electrodes r and 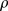 was derived from a random distribution. The recorded time series were filtered in the 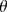 (4–8 Hz) and in the HG (80–180 Hz) frequency. Subsequently, we calculated the HG envelope of the HG time series in each electrode and each trial by taking the absolute value of the Hilbert transform of the filtered time series. The analytic amplitude is a new time series representing the amplitude envelope of the HG-oscillations at any moment in time. We then determined 20 time windows around the stimulus onset each with a width of 500 ms and 400 ms overlap. In order to conduct the TPR permutation test statistic we calculated 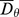 and 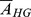 (see above) around each 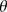 trough in the time window in each electrode and trial which yields 20 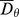 and 20 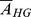 values in each trial from which one 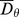 and one 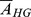 value was randomly chosen in each permutation. In each trial the TPR was calculated from the random 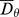 and 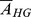 values and correlated (partial correlation) with behavioral measures. In 500 permutation we estimated a distribution of partial correlation which served to assess the significance of the observed partial correlation coefficient. Electrodes exceeding the 95% percentile were considered significantly predictive for performance. In a comparable way learning effects were obtained. In general we tried to find the subset of electrodes within the pool of electrodes which are correlated with performance. Specifically we sought to find those electrodes whose Pearson's correlation coefficient is significantly greater than the partial correlation coefficient (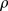). Therefore we again chose randomly one 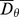 and one 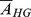 value per trial and correlated (Pearson's correlation – r) the randomly obtained TPR values with behavioral measures. In each permutation we calculated the difference 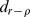 between the randomly obtained 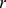 and 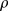. In 500 permutations we estimated a distribution of 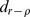 which served to assess the significance of the observed 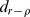. Electrodes exceeding the 95% percentile were considered significantly predictive for performance improvement. Note that this analysis results in spatially more limited clusters than in the ROI-analysis since in the ROI-analysis Pearson's r was used and not the difference of 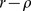.

## Supporting Information

Appendix S1 **Supplementary Material.**
(PDF)

Figure S1 **Prediction of behavior changes as a function of the phase.** Red and black asterisks show the number of signicant electrodes for each of the 20 phase bins for the performance/paCFC correlation and partial correlation, respectively.
(TIF)

Figure S2 **We grouped electrodes into 6 regions of interest.Each outline denotes the grid coverage of one subject.** The bold outline shows the summed coverage across all subjects. The anterior and posterior medial frontal gyrus (FMa, FM), the anterior and the posterior inferior frontal gyrus (FIa, FI), and the superior and inferior sensorimotor cortex (MI,MII). The outline of the grid location of the AMCT participant is given in Figure 5.
(TIF)
