# Supplementary material for: Oscillatory Dynamics Track Motor Performance Improvement in Human Cortex
Source: PLoS One. 2014 Feb 27;9(2):e89576. doi: 10.1371/journal.pone.0089576 (PMC3937444; doi:10.1371/journal.pone.0089576)
Supplement: Appendix S1 — Supplementary Material. (PDF) [file pone.0089576.s001.pdf]

Supplementary Material

Interpretation of partial and Pearson correlation

A geometric interpretation is given by the fact that Pearson correlation, as a similarity measure between random variables, is closely related to the angle between two vectors. The two vectors are the two possible regressions between the two variables. Correlation is high when the angle between the regressions is small. Partial correlation introduces a third variable, in our case time. It measures the angle of the two regression lines in the plane perpendicular to the third variable. Thus, in a 3D space spanned by TPR, performance, and time the plane in which the two possible regressions between TPR and performance can have different orientations with the time axis. If the time axis is orthogonal to the plane, then the correlation between TPR and performance is fully independent of time and unrelated to learning. On the contrary, if the two regression lines are in the same plane as the time axis, then the correlation between TPR and performance is fully time dependent and due to learning. In practice, we can expect to most likely observe mixed effects with regressions oriented between the extremes, indicating mixed contributions of learning and learning unrelated correlations. Importantly, our reasoning is that if we observe a significant Pearson correlation in an electrode that significantly changes if we discount time related correlations (in partial correlation), then the TPR with performance correlation in this electrode is at least partly due to learning related TPR with performance correlations. With this combination of partial and Pearson's correlation we also discount the possibility that the correlation effects are only due to a paCFC variation over time. One prerequisite to conduct the correlation approach described here is that motor performance is linearly independent across trials. We assessed this by estimating the autocorrelation function for the performance measures in each subject. We found the autocorrelation function exceeding the 95 percent confidence interval with a time lag greater than 1 only for the one subject in the AMC task. This is likely a result of the task itself since the subject is required to respond within the same temporal frame. To verify that the significance of the correlation coefficients is not prone to an inter-trial dependency of performance (autocorrelation) we tested in each subject for each electrode showing a significant Pearsons correlation whether it was drawn from a random distribution. In 500 runs we randomly shuffled both the TPR values and performance measures of each trial and calculated the correlation coefficient r. Then we estimated the probability that the observed r values could have been derived from the random distribution. The p-values were all smaller than .002 indicating that the observed r values are not a result of a possible inter-trial dependency of the performance measures.

Stability of coupling phase

The previous analyses showed that performance covaries with paCFC. However, calculating the paCFC at one phase (see Section 4.2) the changes with performance can either be a result of a shift of the HG amplitude peak relative to the θ trough or the result of simultaneous adaption of both frequency components. To test the hypothesis that the covariation is not a result of the HG peak shift we used the fitted phase angle of the trial-wise sine fit functions (see Figure 2) . By means of a linear regression we tested whether there are systematic changes of the phase angle over time. In each subject we compared the slope of the linear regression with an empirical distribution of slopes derived from randomizing the order of trials 500 times. Both for the channels showing correlation and partial correlation no systematic change was found as indicated by slopes within the confidence intervals. This suggests that performance/paCFC covariation is a result of simultaneous adaptation of both frequency components rather than a shift of the HG amplitude peak.

Specificity of θ-HG interaction

We investigated whether HG nested to the θ trough is exclusively predictive for performance. In the three experimental tasks we additionally assessed the specificity of the θ/HG coupling compared to θ/β or θ/γ coupling. For a broad frequency range encompassing β, γ, and high θ range we extracted all frequencies with a band width of 6 Hz and a resolution of 2 Hz; center frequencies: 13-190 Hz). For each sub band ($N_{subbands}$ = 89, 13 belonging to the β band, 21 the γ and 65 to the HG range) we calculated the paCFC with the θ activity and assessed the correlation with behavior for each channel across subjects. The number of electrodes showing a significant ($p < .05$, uncorrected) performance/paCFC correlation varied as a function of frequency band ($N_{\theta/\beta} = 193$, $N_{\theta/\gamma} = 187$, $N_{\theta/H\gamma} = 289$; $\chi^2 = 29.4$; $p < .0001$). The corresponding r-values were compared using a one-way ANOVA with the factor frequency band. The frequency bands predicted behavior differently ($F(2,666) = 10.9$, $p < .0001$). Posthoc tests revealed that the prediction of behavior by the θ/HG coupling was better than the prediction by θ/β coupling or θ/γ coupling (p corrected for Nsubbands < .0001), suggesting a specific effect of the θ/HG coupling for the prediction of motor behavior. Necessity of Interaction We then tested whether θ or HG alone predicted behavior. We compared performance/θ, performance/HG, and performance/paCFC correlation coefficients. As for the specificity analysis, we calculated for each electrode the Pearson's r for correlation of performance with θ, HG, and paCFC and compared r-values of electrodes showing significant correlation. Since Pearsons r is not a metric measure we transformed r values using the inverse hyperbolic tangent

$$\text{arctanh}(r) : \frac{1}{2}\ln\frac{(1+r)}{(1-r)} for|r| < 1 \tag{1}$$

The levels covaried differently with behavior ($F(2,501) = 19.67$, $p < .0001$). Post-hoc tests revealed a significant difference between paCFC and θ ($t(373) = -4{,}81$; $p < .0001$) and paCFC and HG ($t(416) = -4.7$; $p < .0001$) at a Bonferroni corrected significance level. We did not find such difference comparing HG with θ ($p = .5$). Hence, paCFC predicted performance better than θ or HG activity alone. This indicates that the motor performance improvement variation of θ-HG coupling is a result of cooperative modulation of neuronal activity in the two frequency bands rather than the result of a variation of either θ or HG band activity alone.

We next asked whether the trial-by-trial correlation strength depends on the θ phase at which the paCFC is calculated. The previous analysis showed that HG-amplitude varies with the θ phase. Importantly, a variation of performance/paCFC correlation strength as a function of θ phase, could underscore the neurophysiological relevance of paCFC for behavioral adaptation. Therefore, we determined trial-by-trial performance/paCFC correlations at 20 different equally spaced θ phase bins. To test the hypothesis we compared the probability to detect significant electrodes across θ phase bins for correlation and partial correlation. We compared the phase specificity by fitting a cosine function to the probability of significant electrodes (see Figure S1). Both for correlation and partial correlation the fit was highly significant ($p < .001$) with the maximum of significant electrodes close to the trough ($\phi_{correlation} = .4$, $\phi_{partialcorrelation} = .6$). Figure S1 shows the probability of significant correlations both for the performance/paCFC correlation and partial correlation.
