# Supplementary figures and images for: Oscillatory Dynamics Track Motor Performance Improvement in Human Cortex
Source: PLoS One. 2014 Feb 27;9(2):e89576. doi: 10.1371/journal.pone.0089576 (PMC3937444; doi:10.1371/journal.pone.0089576)

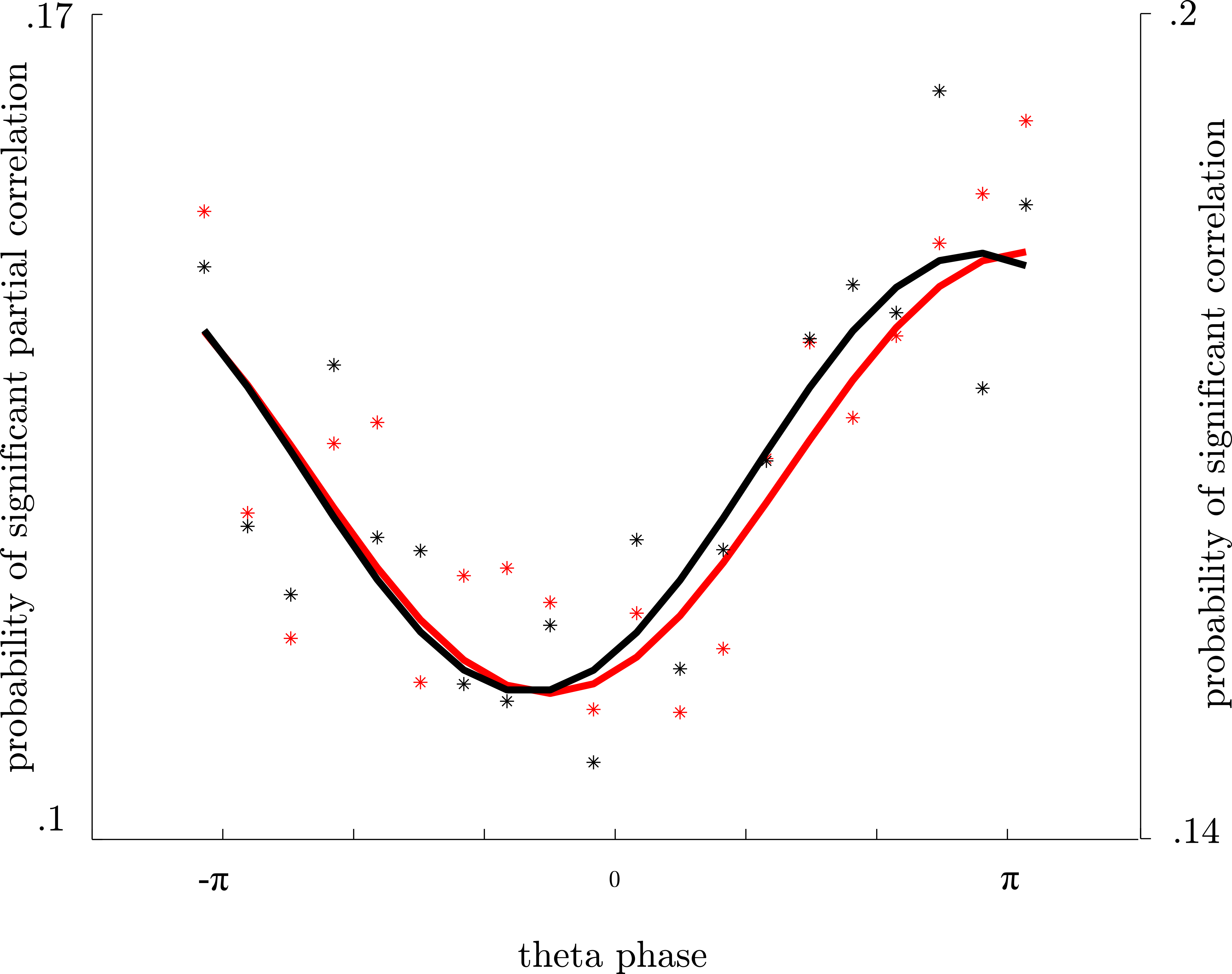

Supplement: Figure S1 — Prediction of behavior changes as a function of the phase. Red and black asterisks show the number of signicant electrodes for each of the 20 phase bins for the performance/paCFC correlation and partial correlation, respectively. (TIF) [file pone.0089576.s002.tif]

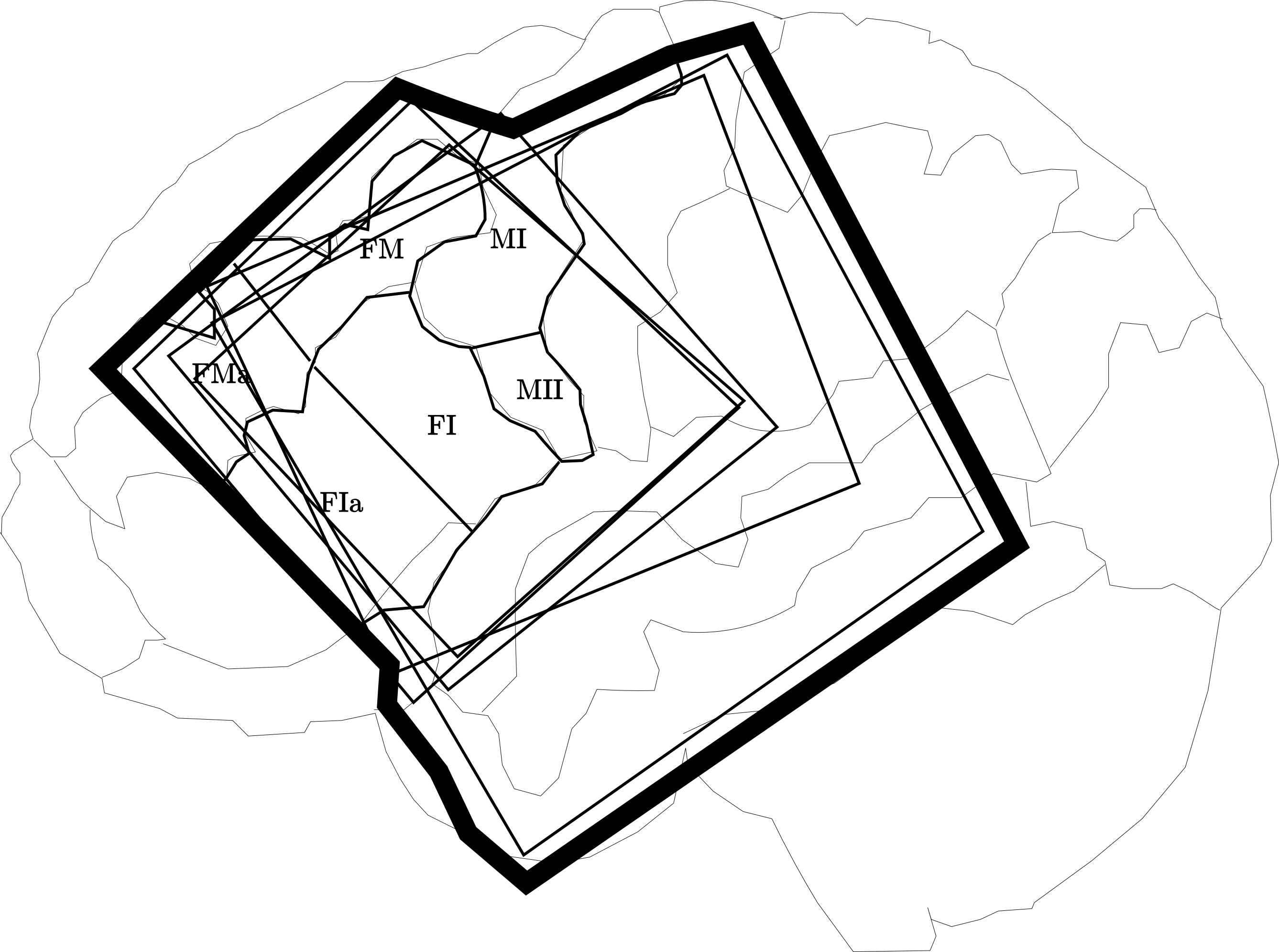

Supplement: Figure S2 — We grouped electrodes into 6 regions of interest.Each outline denotes the grid coverage of one subject. The bold outline shows the summed coverage across all subjects. The anterior and posterior medial frontal gyrus (FMa, FM), the anterior and the posterior inferior frontal gyrus (FIa, FI), and the superior and inferior sensorimotor cortex (MI,MII). The outline of the grid location of the AMCT participant is given in Figure 5. (TIF) [file pone.0089576.s003.tif]
